# Comparative Analysis of Proximate, Nutritional, and Phytochemical Profiles of Soybean Varieties Using FTIR and HPLC

**DOI:** 10.1002/fsn3.71072

**Published:** 2025-10-18

**Authors:** Muhammad Shoaib Fayyaz, Muhammad Farhan Jahangir Chughtai, Adnan Khaliq, Tariq Mehmood, Matteo Bordiga, Emery Lenge Mukonzo Kasongo, Tawfiq Alsulami

**Affiliations:** ^1^ Institute of Food Science and Technology Khwaja Fareed University of Engineering and Information Technology Rahim Yar Khan Pakistan; ^2^ Dipartimento di Scienze del Farmaco Università degli Studi del Piemonte Orientale Novara Italy; ^3^ Land Evaluation and Agro‐Metrology Research Unit, Department of Soil Science, Faculty of Agriculture Research University of Lubumbashi Lubumbashi DR Congo; ^4^ Department of Food Science & Nutrition, College of Food and Agricultural Sciences King Saud University Riyadh Saudi Arabia

**Keywords:** antioxidant activity, FTIR, HPLC, isoflavones quantification, Pakistan soybean varieties, proximate analysis

## Abstract

Soybean is an important legume crop having a highly nutritious profile. This study was performed to compare the phytochemical and nutritional profiles of various soybean varieties cultivated locally in Pakistan along with the quantification of isoflavone aglycones (Daidzein, Genistein, Glycitein) via HPLC and chemical composition analysis through FTIR. Three soybean varieties were selected, i.e., Ajmeri, Willium‐82, and Rawal‐I. The proximate composition of each soybean variety (moisture, fat, protein, and minerals), phytochemical analysis (TPC and TFC), and antioxidant analysis (FRAP and ABTS) of soybean varieties revealed significant variation (*p* < 0.05) in results. Moisture varied from 9.45%–10.45%, protein 33.72%–37.25%, fat 19.04%–21.99%, ash 4.49%–5.55%, and NFE (11.29%–15.14%). Phytochemical analysis revealed TPC in the range 1.35–2.95 (mg GAE/g), TFC 0.44–1.68 (mg CE/g), DPPH 4.21–6.12, FRAP 11.21–14.51, and ABTS 22.15–33.05 (mg TE/g) each with significant variation *p* < 0.05, respectively. HPLC chromatogram showed the presence of isoflavone daidzein ranging from 935.6–515.2 μg/g, glycitein 72.2–62.4 μg/g, and genistein 1071.2–320.4 μg/g in each variety. FTIR spectra of soybean varieties indicated the existence of multiple functional groups seen through the evident stretching of carbon, oxygen, and nitrogen bonds. The lipoxygenase activity in each soybean variety exhibited inhibition potential; maximum inhibition was found in LOX‐2. Overall, the nutritional and phytochemical profile of Ajmeri was found to be better than that of other varieties used in the study.

## Graphical abstract

This study compares the proximate, nutritional, and phytochemical profiles of three soybean varieties (Ajmeri, Willium‐82, and Rawal‐I) using advanced analytical techniques, including HPLC for isoflavone quantification and FTIR for chemical composition analysis. Significant variations were observed in protein, fat, fiber, and antioxidant content, with Ajmeri exhibiting superior nutritional and phytochemical properties. The findings highlight the potential of soybean as a functional food source, offering insights for food processing industries and addressing protein‐energy malnutrition.

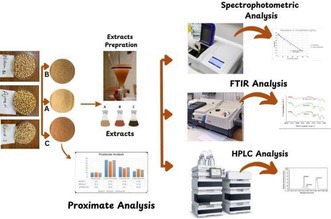

## 1 Introduction

With an approximately 8 billion world population by 2050, a primary concern for food security is to ensure the protein needs of the population can be fulfilled in an affordable and eco‐friendly manner (Saerens et al. 2021). Soybean ( *Glycine max* ) from the pea family Fabaceae is widely being used for developing vegan and protein‐enriched food products. It has been a vegan protein source for human food since the 3rd‐Century BC in China. It contains the highest protein content (35%–42%) compared to other legumes and is a rich source of minerals (4.5%), energy (430 Kcal), and iron (10.5 mg) (Kamboj and Nanda 2018). Soybean contains a significant quantity of vitamins B1 and B2 (0.70 mg and 0.35 mg). All legumes have a fat content below 6% except soybean, which contains 19% fat (Akmalovna 2022).

Soybean contains a number of phenolic compounds, flavonoids, phytates, saponins, and isoflavonoids. Soybean's value is largely based on the potential of isoflavones as they possess cancer‐preventive and antiatherosclerosis properties. Isoflavones in soybean exist in one of the four chemical forms: aglycones, glucosides, malonyl glucoside, and acetylglucoside. Among the others, aglycones including daidzein, genistein, and glycitein exhibit higher bioavailability and health benefits (Zhao et al. 2022). It is worth mentioning that soybean has the potential to control malnutrition in developing and underdeveloped countries, including Pakistan, which has the highest ratio of child malnutrition. According to a report published by the National Nutrition Survey of Pakistan, almost 35% of children in Pakistan are underweight, 45% stunted, 16% wasted, and around 35% are anemic. Keeping in view the aforementioned facts, it is critical to meet the dietary requirements of individuals and provide alternative sources of protein (Asif et al. 2021).

Legumes and legume‐derived foods are of great significance to researchers. Developments in scientific knowledge have led scientists and researchers to develop innovative and alternate foods from natural resources that are both nature‐friendly and cost‐effective. These alternate foods are appealing to consumers owing to their ability to provide a better lifestyle and reduced health disorders. Soybean has been reported to have great potential to serve as an alternate protein source. The health claims of soybean oil and seeds have also been reported in various studies that mainly depend on their physicochemical properties (Qin et al. 2022).

Owing to the therapeutic significance of soybean, food processing industries are primarily focusing on developing innovative soybean products having excellent functional and nutritional value (Khosravi and Razavi 2021). Furthermore, consumer demands for lactose‐free and vegan foods have increased over the past few decades, indicating the potential of soybean in this regard. Pakistan is naturally blessed with a diverse climate and exceptional growth conditions, i.e., soil, water, irrigation, and fertilizers. Presently, the cultivation of soybean is concentrated in the Punjab province of Pakistan. The crop is well suited to climatic conditions, giving a yield of 1.50–1.75 tons per acre (Raza et al. 2023). The production status of soybean, along with its challenges and prospects, is well cited in the literature; however, an in‐depth analysis of indigenously grown soybean cultivars in Pakistan has not been conducted so far. The present research has been performed to characterize the nutritional profiles of soybean cultivars via HPLC and FTIR, which revealed significant variations in the phytochemical and nutritional profiles of these varieties. The data obtained may serve as a useful tool in selecting a suitable soybean variety for food processing industries.

## 2 Materials and Methods

**FIGURE 1:**
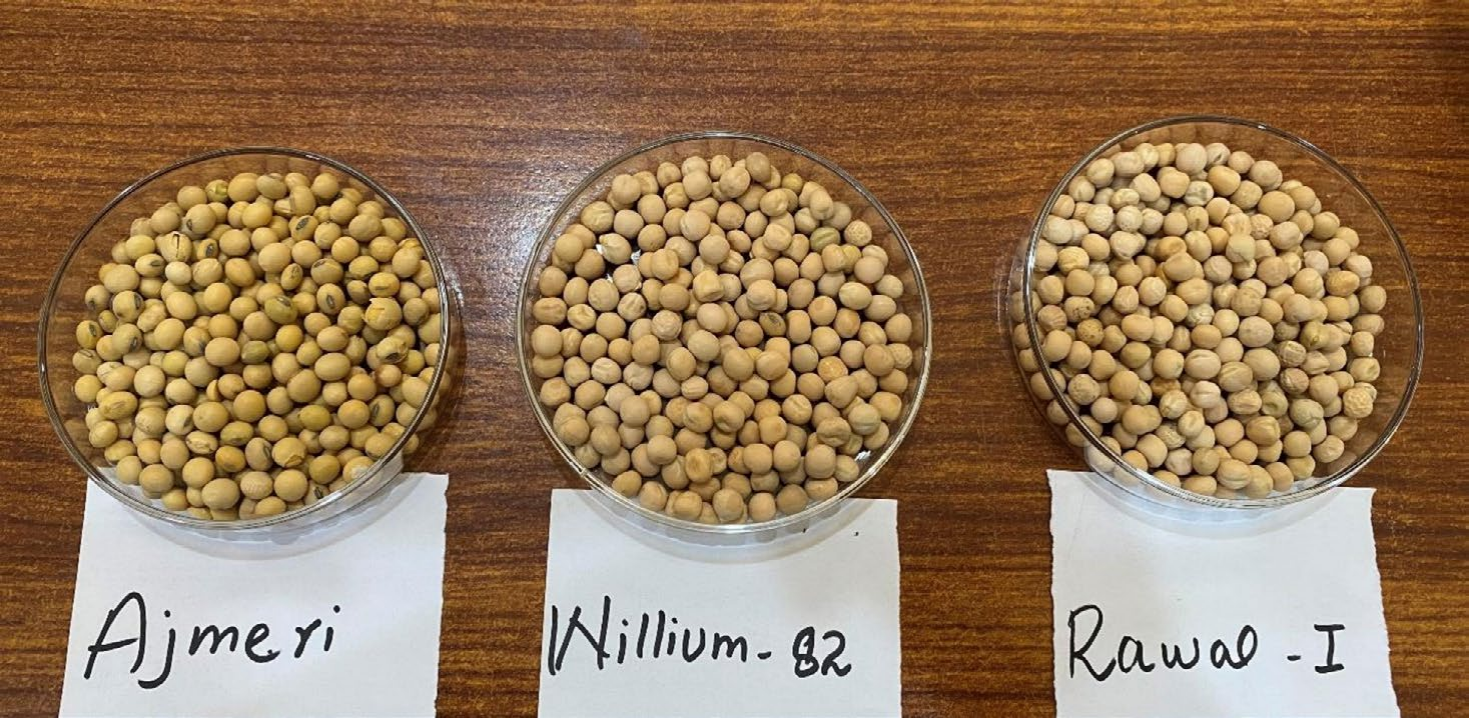
Soybean varieties Ajmeri, Rawal‐I and Willium‐82.

Soybean varieties including Ajmeri, Rawal‐I, and Willium‐82, as shown in Figure 1, were obtained from NARC Islamabad and AARI Faisalabad. Each soybean variety was analyzed in triplicates for the following methods. The chemicals employed in this research were procured from Sigma Aldrich (USA), Merck (Germany), and DaeJung (South Korea).

### 2.1 Compositional Analysis

The proximate conformation of soy flour from each variety was done in terms of moisture, fat, protein, fiber, minerals, and nitrogen‐free extract (NFE) in accordance with the protocol outlined in (AOAC 2016).

### 2.2 Mineral Analysis

Minerals including Fe, Mn, Mg, and Zn were determined through Atomic Absorption Spectrophotometer (AAS model 240) and Flame Photometer (Model 410) as described in (AOAC 2016).

### 2.3 Lipoxygenase Activity (LOX)

Lipoxygenase enzyme, naturally present in soybean, initiates lipid oxidation leading to the production of H_2_O_2_. The lipoxygenase activity of soybean varieties was evaluated using a UV–VIS spectrophotometer (Model AA240) at 234 nm for LOX‐1 and 2, whereas LOX‐3 was determined at 280 nm. The defatted soy flour samples were treated with the substrate linoleic acid. 1 g of defatted soy flour from each variety was extracted using 50 mL of buffer solution containing sodium phosphate having a pH of 6.8 and a concentration of 0.2 M. The process was continued for 1.5 h in an orbital shaker. Centrifugation at 15,000 rpm for 10 min was performed, and the lipoxygenase activity was measured through the supernatant. The absorbance potential was increased by 2.0/min due to enzymatic activity, and the observations were recorded at 0, 2, 4, 6, and 8 min intervals. The results were expressed as absorbance per minute (González‐Gordo et al. 2023).

### 2.4 Phytochemical Screening

Phytochemical screening for each soybean variety in terms of phenolics (TPC) and flavonoids (TFC) was performed using a UV–VIS spectrophotometer.

### 2.5 Sample Preparation

Soybeans were milled and 5 g soy flour was taken in a test tube. The flour was mixed under sonication for 25 min with aqueous acetone having a purity of 70% and a volume of 50 mL at room temperature. The mixture was then vacuum filtered and kept in refrigeration until assayed.

Total phenolics in each soybean variety were assessed using the Folin–Ciocalteu procedure as described by Ma et al. (2022). Aliquots of aq. acetone extract (0.1 mL) were taken in test tubes. Distilled water was added in test tubes to make the volume up to 0.5 mL. Then 20 mL Folin's reagent was added, followed by the addition of aq. Na_2_CO_3_ (1.25 mL). Test tubes were then vortexed, and the absorbance was measured after 30 min at 720 nm against a blank. The blank solution comprised merely the extraction solvent (0.1 mL). The concentration of TPC was measured as gallic acid equivalent from the curve of standard solutions.

TFC of each soybean variety was measured using a calorimetric method. The standard solution of catechin was used as a reference. Extracted samples and standards were vortexed with distilled water and 5% sodium nitrate solution (1250 and 75 μL, respectively). The mixture was kept aside for 10 min, followed by the addition of 1 M sodium hydroxide solution and distilled water (500 and 275 μL, respectively). The mixture's absorbance was measured at 510 nm immediately, and the results were expressed as mg CAE/g (Choi et al. 2023).

### 2.6 Isoflavones Determination Through High Performance Liquid Chromatography (HPLC)

#### 2.6.1 Reagents

HPLC grade reagents including water and acetonitrile were procured from Sigma Aldrich (USA) and Merck (Germany).

#### 2.6.2 Instrument and HPLC Conditions

The analysis was performed using an Agilent 1200 series HPLC model equipped with a quaternary pump and a photo diode array detector. The instrument contained an ODS AM 303 column having specifications of 5 μm; 4.6 × 250 mm covered with a C18 insert column Nova‐Pak. The chemical profile of each soybean variety (Ajmeri, Willium‐82, and Rawal‐I) within a sample varied owing to its seed size and maturity. Therefore, the chromatogram of each variety in a single sample was averaged, resulting in one spectrum.

The extraction of isoflavones was done according to the method described by (Cho et al. 2020) with partial modification. Soybean seeds were ground and homogenized. To enhance the extraction of isoflavones, 5 g soybean powder was extracted with 100 mL distilled water using an ultrasonic probe (Elmasonic Easy 30 H, Singen, Germany) for 10 min (Leksawasdi et al. 2022). 1 g sample was hydrolyzed with 0.1 N HCl and was kept for 2 h at 100°C followed by the addition of 10 mL methanol. The solution was filtered using a Whatman No. 42 filter paper. Supernatants were separated and stored at 4°C until further use. Isocratic elution was performed at 0.8 mL/min flow rate using 30% (v/v) methanol in water solution and the absorbance was recorded at 254 nm.

### 2.7 Fourier Transform Infrared Spectroscopy (FTIR)

Soybean seeds were ground and 30 g of powdered sample was separated, which was further passed through a sieve having mesh size 60 and opening at 250 μm. The spectral analysis of soybean seed and powder was done using an FTIR spectrometer (USA model Nicolet 6700 series). The instrument, equipped with an attenuated total reflectance (ATR) sampling mode, showed spectral data of soybean seed and powder. The absorbance spectra of each soybean variety were collected in MIR wavelength ranging from 2500 to 25,000 nm (4000–400 cm^−1^) at an interval of 4 cm^−1^ spectral resolution (Amanah et al. 2022). The average spectrum of each soybean variety (Ajmeri, Willium‐82, and Rawal‐I) was obtained for further interpretation.

### 2.8 Antioxidant Potential of Soybean

Antioxidant potential of soybean was determined using a modified DPPH assay. The scavenging potential contributes to the non‐enzymatic antioxidant activity. Aliquots (0.5 mL) from extracts were mixed with an alcoholic DPPH solution of 0.5 mmol and acetate buffer solution of 100 mmol having pH 5.5 (0.25 and 0.5 mL, respectively). The solution was kept aside for 30 min, and the absorbance was measured at 517 nm against a blank. The blank solution comprised absolute ethanol (0.5 mL) (Choi et al. 2020).

### 2.9 FRAP Assay

The antioxidant potential of each soybean variety in terms of Ferric reducing antioxidant power was determined using the spectroscopic analysis. Change in absorbance was measured at 595 nm on a UV–VIS spectrophotometer (model AA240, Varian). The reagents used were 2,4,6 Tripyridyltriazine (10 mM), ferric acid solution (20 mM) and 0.3 M acetate buffer having pH 3.6. The ferric reduction potential of the sample was described as mgTE/g (Choi et al. 2020).

### 2.10 ABTS Assay

The antioxidant potential of each soybean variety in terms of ABTS (2,2‐azinobis(3‐ethylbenzothiazoline‐6‐sulfonic acid) was determined using a UV–Vis spectrophotometer. Diluted ABTS solution (100 μL) and soybean sample (10 μL) were coated in 48‐well microplates. The difference in absorbance was measured at 734 nm on a UV–Vis spectrophotometer (Model AA240, Varian) at an interval of 15 s. A calibration curve was obtained using Trolox solution and the results were described as mg TE/g (Chu et al. 2021).

### 2.11 Statistical Analysis

The readings for each parameter were obtained in triplicate, and the data were subjected to statistical analysis. One‐way ANOVA under CRD, confidence interval 95%, and post hoc HSD for comparison of mean values were applied with significance *p* < 0.05 using Statistix software version 8.1 (Awan et al. 2018).

## 3 Results and Discussion

### 3.1 Compositional Analysis

The compositional analysis of three soybean varieties revealed significant differences (*p* < 0.05) in moisture, fat, protein, ash, and fiber content as shown in Table 1. The moisture content for Ajmeri, Rawal‐I, and Willium‐82 was 9.65 ± 0.46, 10.15 ± 0.35, and 9.45 ± 0.45, respectively. Variations in moisture content may be due to differences in agronomic practices of the varieties, moisture concentration during harvest, and processing conditions. Previously, the moisture content in soybean has been reported as 9%–11% by Kudełka et al. (2021), 8.76%–9.32% by Mitharwal and Chauhan (2022), and 9.15% by Guo et al. (2023), respectively. These findings are in line with the present findings.

**TABLE 1:** Proximate composition of soybean varieties.

Varieties	Moisture (%)	Crude protein (%)	Crude fat (%)	Crude fiber (%)	Ash (%)	NFE (%)
Ajmeri	9.65 ± 0.46^b^	37.25 ± 1.50^a^	21.99 ± 1.03^a^	12.98 ± 0.55^c^	5.55 ± 0.25^a^	12.58 ± 1.19^c^
Rawal‐I	10.15 ± 0.35^a^	33.72 ± 1.55^cd^	19.04 ± 0.71^b^	17.46 ± 0.85^a^	4.49 ± 0.2^c^	15.14 ± 2.21^a^
Willlium‐82	9.45 ± 0.45^ab^	36.76 ± 1.65^ab^	21.3 ± 0.85^a^	15.95 ± 0.75^ab^	5.25 ± 0.21^ab^	11.29 ± 1.15^b^

*Note:* All values are in terms of mean ± SD of means; *n* = 3 sets; ^abcd^Means in columns with different superscripts differ (*p* < 0.01).

Abbreviation: NFE, nitrogen‐free extract.

The analysis revealed the highest protein content in Ajmeri (37.25% ± 1.50%) followed by Willium‐82 (36.76% ± 1.65%) and Rawal‐I (33.72% ± 1.55%). Soybean has been blessed with an impressive protein profile naturally. Protein content in soybean has been reported previously in the range 40.65%–44.58% (Anwar et al. 2016). In another study, the protein content in soybean was found to be in the range 44.51%–46.45% (Abubakar et al. 2014). The variation in protein content might be due to the genetic differences among varieties. Protein content in soybean is a quantitative trait influenced by various genes, as indicated in a study conducted by Chen et al. (2017). Environmental factors also play a significant role in this cause. Obua et al. (2021) found that regions with higher temperatures and lower rainfall during the bean formation phase result in an increased protein content in soybeans. In another study, research in the southeastern region of the United States revealed that management practices during the planting of soybean seeds can modulate protein levels owing to environmental conditions (Epie et al. 2023). The protein content in soybeans has also been reported as 35.05%–38.11% and 34.15% (de Barros et al. 2014; Lee et al. 2013), respectively, which are in line with the present findings. Varieties with such high protein content are preferred for products like protein powders, tofu, and meat analogs. Superior protein‐containing varieties may have better gelation properties and water‐holding capacity. These factors are crucial in the development of products like yogurt and textured vegetable proteins. However, regional variability and accessibility to such varieties might be a challenge in this regard.

The finding revealed significant variations in fat content. The highest concentration of fat was found in Ajmeri (21.99% ± 1.03%) followed by Willium‐82 (21.3% ± 0.85%) and the least crude fat was recorded in Rawal‐I (19.04% ± 0.71%). These results are supported by the findings of various researchers. The crude fat in soybean was found in the range of 16.85%–19.45%, 19.53%–23.01%, and 16.21%–21.15% as reported by (Jarecki and Bobrecka‐Jamro 2021; Sharma et al. 2014), respectively.

Crude fiber content in each variety also showed significant variation; i.e., maximum crude fiber was recorded in Rawal‐I (17.46% ± 0.85%), followed by Willium‐82 (15.95% ± 0.75%), and minimum fiber content was found in Ajmeri (12.98% ± 0.55%). Fiber content in food products is considered a functional food ingredient owing to its therapeutic effect against cancer and cardiovascular diseases. The fiber content in soybean varied from 10.45% to 14.50% as reported by Shurtleff and Aoyagi (2016) and 11.5% to 13.75% as reported by Jansone et al. (2021), respectively. The observations of the current research are supported by Mitharwal and Chauhan (2022).

The concentration of ash (minerals) in foods is important for food processing industries; the maximum concentration of ash was recorded in Ajmeri (5.55% ± 0.25%) followed by Willium‐82 (5.25% ± 0.21%) and the minimum ash was found in Rawal‐I (4.49% ± 0.2%). Anwar et al. (2016) observed the ash content in soybean in the range of 5.45%–6.85%. Similar results were also reported by Staniak et al. (2021) who found the ash content in soybean as 3.85%.

Consequently, the maximum nitrogen‐free extract (NFE) was found in Rawal‐I (15.14% ± 2.21%), followed by Ajmeri (12.58% ± 1.19%), and the least NFE was found in Willium‐82 (11.29% ± 1.15%). NFE corresponds to the concentration of carbohydrates including sugars. Current observations are in accordance with Mitharwal and Chauhan (2022). They found NFE in soybean in the range of 15.41%–21.65%.

### 3.2 Mineral Profiling

Mineral profile of each soybean variety showed significant variation (*p* < 0.05) regarding the presence of minerals such as Zn, Mn, Mg, Fe, Ca, Na, and K. Table 2 shows the concentration of various minerals found in soybean. The content of various minerals found in Ajmeri was: K 1975.15 ± 35.25, Mg (375 ± 6.50), Ca (270 ± 4.54), Fe (22.25 ± 0.65), Zn (5.54 ± 1.00), Na (4.25 ± 0.15), and Mn (3.68 ± 0.11) mg/100 g, respectively. Overall, the trend observed for minerals in each variety was Mn < Na < Zn < Fe < Ca < Mg < K. Variation in minerals might be due to soil fertility and agronomic conditions adopted for the growth of soybean varieties (Kim et al. 2021). Also, it can be due to crop rotation and mineral exhaustion due to repetitive sowing of the same crop in the fields (Chen et al. 2015). Jiao et al. (2012) observed the soybean's mineral composition grown in the United States. They reported potassium content in soybean varied from 1515.25 ± 38.15 to 1645.54 ± 23.56 mg/100 g, Ca from 122.15 ± 6.65 to 224.95 ± 7.40, and Zn from 2.09 ± 0.05 to 3.65 ± 0.19 mg/100 g, respectively. The composition of soybean was also investigated by Özcan and Al Juhaimi (2014). They reported potassium mineral was found in maximum concentration in soybean as compared to other minerals, varying from 1625.4 mg to 2045.5 mg. The minerals composition of the present research aligns with the findings of Costa et al. (2015), who found manganese as 1.78 mg and Zinc as 5.25 mg per 100 g of soybean. This variation in mineral profile could be due to various reasons including geographical differences and certain biological factors. Also, the genetic makeup of each soybean variety plays a vital role in this regard (McClain et al. 2018).

**TABLE 2:** Mineral composition of soybean varieties.

Varieties	K	Fe	Mg	Ca	Na	Zn	Mn
Ajmeri	1975.15 ± 35.25^a^	22.25 ± 0.65^b^	375 ± 6.50^a^	270 ± 4.54^a^	4.25 ± 0.15^c^	5.54 ± 1.35^a^	3.68 ± 0.11^b^
Rawal‐I	1615.25 ± 31.95^b^	11.23 ± 1.10^c^	256.35 ± 5.15^c^	175.40 ± 3.45^a^	3.15 ± 1.22^c^	4.95 ± 0.81^b^	2.20 ± 0.45^a^
Willium‐82	1845.25 ± 36.25^b^	17.56 ± 1.12^a^	412.51 ± 8.22^b^	310.36 ± 4.15^b^	4.26 ± 0.56^b^	4.95 ± 2.21^c^	2.88 ± 1.12^c^

*Note:* All values are in terms of mean ± SD of means; *n* = 3 sets; ^abcd^Means in columns with different superscripts differ (*p* < 0.01).

### 3.3 Lipoxygenase‐1 Activity

**FIGURE 2:**
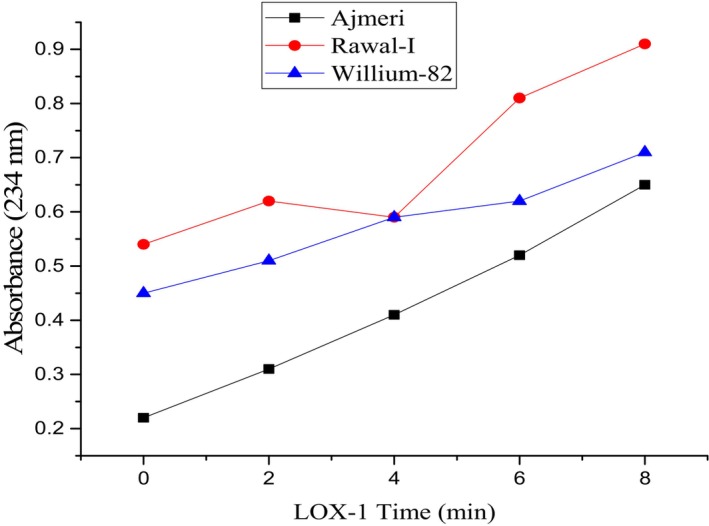
Lipoxygenase (LOX)‐1 potential of soybean varieties at 234 nm registered at pH 9.

Statistical analysis depicting the LOX‐1 activity of each soybean variety showed significant variation in results. LOX‐1 activity of Ajmeri, Rawal‐I, and Willium‐82 is shown in Figure 2. It is evident from the graph that the mean value of absorbance increased with time, revealing the LOX‐1 activity of the varieties. The minimum value of absorbance was observed at 0 min in Ajmeri as 0.29 ± 0.005, and the maximum value was recorded in Rawal‐I (0.28 ± 0.004) at the same time. At a 6‐min interval, the maximum value was observed in Rawal‐I as 1.35 ± 0.015, and the least value was found in Ajmeri as 0.67 ± 0.012. Minimum absorption was observed in Ajmeri, followed by Rawal‐I and Willium‐82, which shows Ajmeri possesses less LOX‐1 activity and peroxidation potential as compared to other varieties.

### 3.4 Lipoxygenase‐2 Activity

**FIGURE 3:**
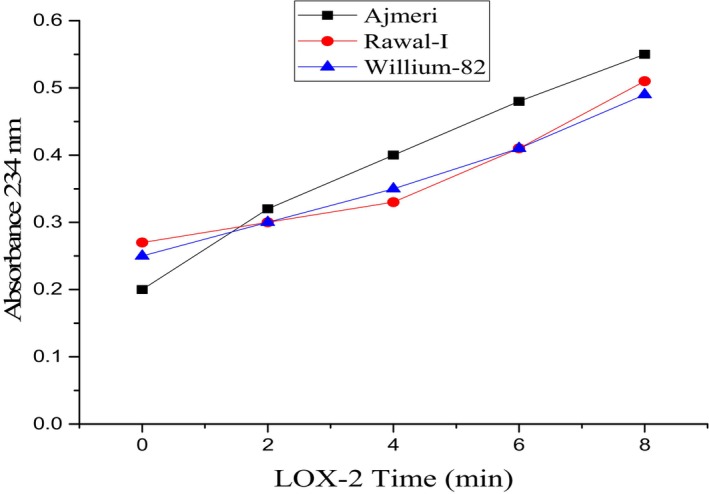
Lipoxygenase (LOX)‐2 potential of soybean varieties at 234 nm registered at pH 6.8.

The impact of LOX‐2 activity showed significant variations among the soybean varieties. Figure 3 shows the absorbance potential of soybean varieties with time against the LOX‐2 activity. It is evident from the Figure that the minimum value was observed in Ajmeri at 0.15 ± 0.0021 and maximum in Rawal‐I at 0.25 ± 0.0017 at the same time (0 min). However, the value of absorbance increased with time along with peroxidation potential. Overall, the trend for LOX‐2 activity was found as Rawal‐I > Ajmeri > Willium‐82 as 0.31, 0.28, and 0.21, respectively.

### 3.5 Lipoxygenase‐3 Activity

**FIGURE 4:**
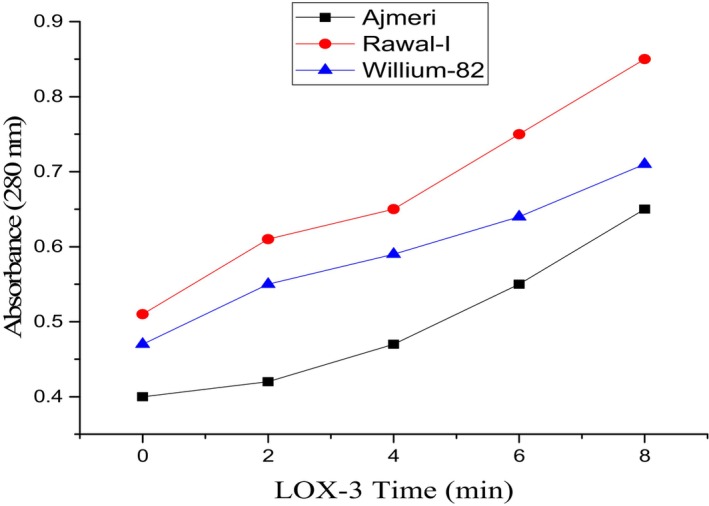
Lipoxygenase (LOX)‐3 potential of soybean varieties at 280 nm registered at pH 7.1.

Soybean varieties showing LOX‐3 activity are shown in Figure 4. LOX‐3 activity showed the best results at pH 7.0 with the least peroxidation potential and production of ketodiene. The results for LOX‐3 activity are in accordance with the former LOX activities with a similar trend, i.e., LOX activity increased with time from 0 to 8 min. The values for LOX‐3 varied from 0.38 ± 0.005 to 0.65 ± 0.02 in Ajmeri from 0 to 6 min interval, followed by Willium‐82 and Rawal‐I.

### 3.6 Isoflavone Through HPLC

**FIGURE 5:**
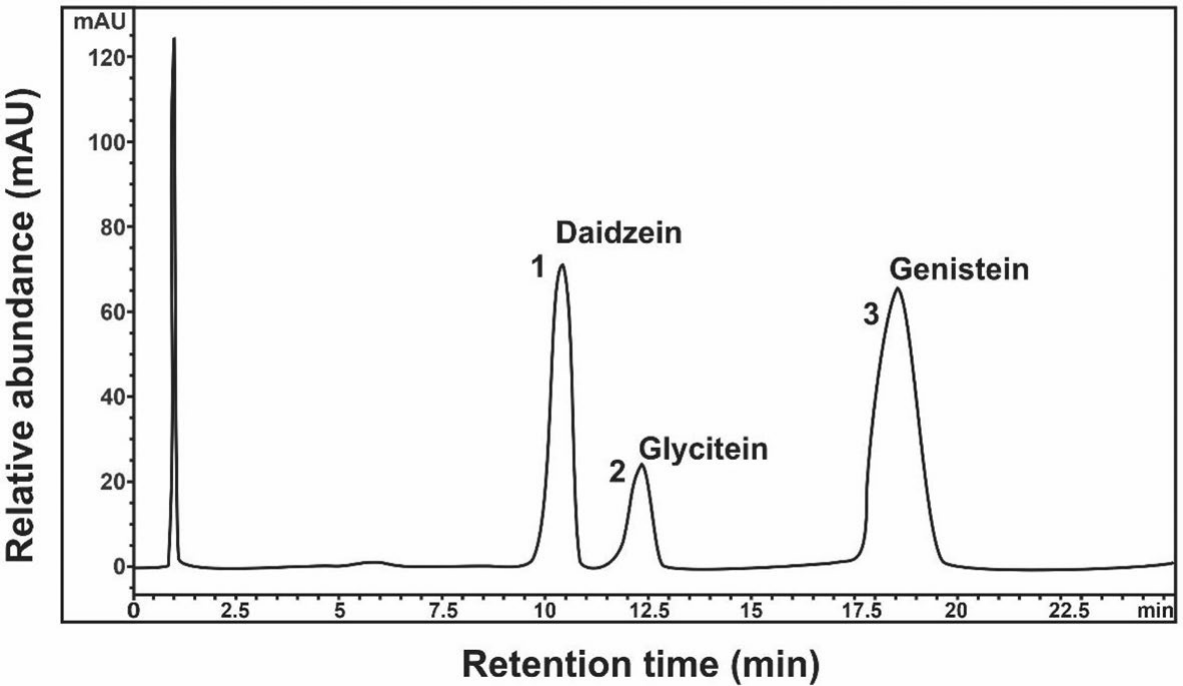
HPLC chromatogram of hydrolyzed extract of soybean at 254 nm. HPLC, high‐performance liquid chromatography.

Among soybean isoflavones, genistein derivatives are the predominant ones. Three prominent isoflavones observed through this analysis included genistein, daidzein, and glycitein, as shown in Figure 5. Isoflavone aglycone derivatives have promising results in preventing human diseases, including cardiovascular disorders and cancer (Cho et al. 2020). The supplementation of soy isoflavones has also been reported to reduce both systolic and diastolic blood pressure (Lei et al. 2024). The consumption of soy protein containing isoflavones exhibited a positive impact against aging and skin hydration in postmenopausal women (Rizzo et al. 2023). Therefore, the contents of the aforementioned isoflavone aglycones were quantified in the present study from hydrolyzed soybean extracts as follows: (1) *y* = 139.13*x* – 120.14, *r* ^2^ = 0.9999; (2) *y* = 92.5965 – 3.82, *r* ^2^ = 0.9998; (3), *y* = 181.13*x* – 129.20, *r* ^2^ = 0.9999, where *y* = peak area, *x* = conc. of the std. (μg/mL). The concentration of each aglycone was determined on the basis of peak areas in the chromatogram. Isoflavone content showed significant variation among varieties. Table 3 shows the individual concentration of each aglycone in soybean varieties along with mean values. It is evident from Table 3 that the mean value for daidzein was found to be maximum in Ajmeri (935.5 μg/g), followed by Willium‐82 and Rawal‐I. Glycitein exhibited maximum concentration in Ajmeri (72.2 μg/g), followed by Willium‐82 and Rawal‐I. A similar trend was observed for genistein in each of the three soybean varieties. The mean value for overall isoflavone content was found to be highest in Ajmeri (693 ± 541.9), followed by Willium‐82 (466 ± 381.6) and Rawal‐I (219.2 ± 151.3), respectively. These results are in accordance with the findings of Megha et al. (2024). Generally, isoflavone content is affected by the genotype, agronomic practices, and the interaction of these factors. The environment and genotype had a great effect on the isoflavone content, as reported by Kassem (2021) and Wang et al. 2022).

**TABLE 3:** Isoflavone contents of soybean varieties quantified through high‐performance liquid chromatography (HPLC).

Variety	Isoflavone contents in soybean varieties			
	Daidzein (μg/g)	Glycitein (μg/g)	Genistein (μg/g)	Mean ± SD
Ajmeri	935.6	72.2	1071.2	693 ± 541.9
Rawal‐I	292.4	45.5	320.4	219.2 ± 151.3
Willium‐82	515.2	62.4	820.9	466 ± 381.6
Mean ± SD	581.1 ± 326.6	60 ± 13.5	737.5 ± 382.2	

*Note:* *n* = 3 sets.

Abbreviation: SD, standard deviation.

### 3.7 Spectral Interpretation of FTIR

**FIGURE 6:**
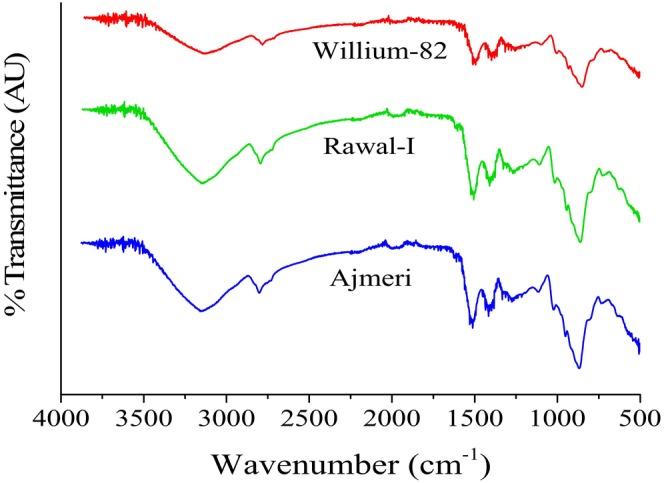
Fourier‐transform infrared spectroscopy spectra of soybean varieties depicting various functional groups.

The FTIR spectra of soybean varieties is shown in Figure 6. The spectra depicted the presence of various functional groups associated with the main components including proteins, lipids, and carbohydrates. The spectra, as shown in Figure 6, can be broadly classified into two waveband ranges: group frequencies and molecular fingerprint frequencies. In the first fingerprint range of 500–1500 cm^−1^, the stretching of carbon–oxygen and carbon–carbon bonds can be identified. The stretching vibration of carbon–nitrogen molecules, as in proteins, can be seen at 1100 cm^−1^ and the stretching of fatty acid molecules (CH2 and CH3) can be seen in the band range of 1200–1500 cm^−1^ (Amanah et al. 2020; Jung et al. 2020). Each soybean variety showed stretching of functional groups owing to its capacity. The graph indicated the presence of various functional groups in soybean contributing to its high nutritional value.

### 3.8 Phytochemical Analysis

Phytochemical analysis of soybean varieties is shown in Table 4. It was observed that maximum phenolic and flavonoid contents were observed in Ajmeri, i.e., 2.95 mgGAE/g and 1.68 mgCE/g, respectively, followed by Willium‐82 and Rawal‐I. Phenolic acids account for 27 to 70% of total phenolic contents in soybean (Yu et al. 2021). Phenolic compounds have widely been studied for their physiological benefits inside the body, including anti‐carcinogenic and anti‐oxidant properties (Cabezudo et al. 2021). The findings for total phenolics in soybean are in accordance with Josipović et al. (2016). They documented TPC and TFC of different soybean cultivars. Results revealed variation from 2.15 to 3.21 mgGAE/g for TPC and 0.40 to 0.65 mg CAE/g for TFC. Choi et al. (2021) investigated total phenolic compounds in colored soybean seeds. They found TPC in the range of 2.58 ± 0.41 mgGAE/g of dry weight in yellow seeds.

**TABLE 4:** Phytochemical analysis of soybean varieties.

Varieties	TPC (mg GAE/g)	TFC (mg CE/g)	DPPH (mg TE/g)	FRAP (mg TE/g)	ABTS (mg TE/g)
Ajmeri	2.95 ± 0.02^a^	1.68 ± 0.02^c^	6.12 ± 0.12^b^	14.51 ± 0.55^bc^	30.35 ± 1.56^bc^
Rawal‐I	1.35 ± 0.01^b^	0.44 ± 0.03^b^	5.85 ± 0.22^c^	11.21 ± 0.22^a^	28.56 ± 2.01^c^
Willium‐82	2.45 ± 0.03^c^	1.12 ± 0.03^a^	4.21 ± 0.19^a^	13.75 ± 0.35^b^	22.15 ± 2.11^ab^

*Note:* All values are in terms of mean ± SD of means; *n* = 3 sets; ^abc^Means in columns with different superscripts differ (*p* < 0.01).

Abbreviations: ABTS, 2,2′‐azino‐bis (3‐ethylbenzothiazoline‐6‐sulfonic acid) radical scavenging activity; CE, catechin equivalent; DPPH, 2,2‐diphenyl‐1‐picrylhydrazyl radical scavenging activity; FRAP, ferric reducing antioxidant power; GAE, gallic acid equivalent; TE, trolox equivalent; TFC, total flavonoid content; TPC, total phenolic content.

Soybean varieties were also subject to antioxidant analysis, including DPPH. Complete results depicting the antioxidant potential of each soybean variety are shown in Table 4. The maximum DPPH results were shown by Ajmeri (5.45 ± 0.15 mgTE/g), followed by Willium‐82 (5.25 ± 0.11 mgTE/g) and Rawal‐I (4.41 ± 0.12 mgTE/g), respectively. Consumption of antioxidants in diets has proven results against various degenerative disorders in the body (Nemzer et al. 2022). The DPPH findings for antioxidant potential in the present study are consistent with the findings of Prvulović et al. (2016). They investigated the DPPH potential of soybean varieties, and the results ranged from 3.71 ± 0.04 to 6.98 ± 0.06 mgTE/g. Chung et al. (2011) found DPPH in the range of 0.7–2.1 mmoles TE/g in yellow soybean seeds, and found DPPH in fermented soy flour in the range of 3.95–9.94 μmol TE/g. Soybean has been reported to possess high antioxidant properties, making it a suitable food for nutritional purposes.

Similar trends were observed for the FRAP assay. The maximum mean value for the FRAP assay was obtained for Ajmeri (13.15 ± 0.85), followed by Willium‐82 (12.17 ± 0.75) and Rawal‐I (11.30 ± 0.69). The trend for the FRAP assay is similar to the trend obtained for DPPH. These findings are in accordance with the results of (Khosravi and Razavi 2021; Kulprachakarn et al. 2021). In the present research, the maximum ABTS value was observed in Ajmeri (29.15 ± 2.01), followed by Willium‐82 and Rawal‐I, i.e., 26.95 ± 1.89 and 22.29 ± 1.45, respectively.

## 4 Conclusion

The research was performed using three cultivars of soybean grown locally in Pakistan. The samples were procured from NARC Islamabad and AARI Faisalabad. The objectives of using the samples and performing the analysis were to assess and compare the proximate, nutritional, and phytochemical profiles of these soybean varieties. Soybean, owing to its rich protein content, can be used as an alternate source of protein to combat protein energy malnutrition in Pakistan. The crop can also be used as a functional food to combat various physiological disorders due to its high phytochemical profile. The varieties used in this research revealed significant variation among the tested components. Among the three varieties, Ajmeri was found to possess maximum nutritional capacity and also had the potential to maintain seed quality. A variety with such high protein content is recommended for the development of protein‐enriched products such as tofu and meat analogs. The nutritional profiling of the various soybean cultivars through this study is an index of its nutritional worth and potential to be used as a functional and alternative protein food. The crop can be well utilized to combat protein energy malnutrition (PEM) in Pakistani communities who are unable to afford high‐quality protein sources (such as meat) due to their limited economic conditions. Soybean can also be processed and used in the development of meat analogs, which is not only the trending consumer demand but also is healthy due to its cholesterol‐free nature. However, much remains to be explored in this domain as the processing of soybean through extrusion is a challenge due to its high lipid content, which can be controlled via enzymatic inactivation, high‐pressure processing, and degumming.

## Conflicts of Interest

The authors declare no conflicts of interest.

## Data Availability

The data that support the findings of this study are available on request from the corresponding author.

## References

[fsn371072-bib-0001] Abubakar, A. , S. Ibrahim , and F. I. J. Musa . 2014. “Physicochemical Analysis of Soxhlet Extracted Oils From Selected Northern Nigerian Seeds.” International Journal of Biological and Life Sciences 8, no. 11: 1174–1177.

[fsn371072-bib-0002] Akmalovna, A. C. 2022. Biological Properties of Soybean (Conference Presentation). E Conference Zone, 90–94.

[fsn371072-bib-0003] Amanah, H. Z. , R. Joshi , R. E. Masithoh , M. G. Choung , K. H. Kim , and G. Kim . 2020. “Nondestructive Measurement of Anthocyanin in Intact Soybean Seed Using Fourier Transform Near‐Infrared (FT‐NIR) and Fourier Transform Infrared (FT‐IR) Spectroscopy.” Food Technology 111: 103477. 10.3390/foods11020232 PMC8774574 35053964

[fsn371072-bib-0004] Amanah, H. Z. , S. S. Tunny , R. E. Masithoh , et al. 2022. “Nondestructive Prediction of Isoflavones and Oligosaccharides in Intact Soybean Seed Using Fourier Transform Near‐Infrared (FT‐NIR) and Fourier Transform Infrared (FT‐IR) Spectroscopic Techniques.” Food 11, no. 2: 232. 10.3390/foods11020232 PMC8774574 35053964

[fsn371072-bib-0005] Anwar, F. , G. M. Kamal , F. Nadeem , and G. Shabir . 2016. “Variations of Quality Characteristics Among Oils of Different Soybean Varieties.” Journal of King Saud University, Science 28, no. 4: 332–338.

[fsn371072-bib-0006] Asif, M. , H. Rahman , A. Aziz , M. Adnan , A. Ali , and M. E. Safdar . 2021. “Seed Priming Improves Growth, Yield and Seed Oil Contents of Soybean ( *Glycine max* L.) Cultivars Under Semi‐Arid Conditions of Sargodha, Pakistan.” Pakistan Journal of Biochemistry and Molecular Biology 22, no. 5–6: 27–37.

[fsn371072-bib-0007] Awan, K. A. , M. S. Butt , M. K. Sharif , and F. Hussain . 2018. “Compositional Profiling of Selected Pakistani Date Cultivars.” Pakistan Journal of Agricultural Sciences 55, no. 3: 575–581.

[fsn371072-bib-0008] Cabezudo, I. , M. R. Meini , C. Di Ponte , N. Melnichuk , C. E. Boschetti , and D. Romanini . 2021. “Soybean (*Glycine max*) Hull Valorization Through the Extraction of Polyphenols by Green Alternative Methods.” Food Chemistry 338: 128131. 33091982 10.1016/j.foodchem.2020.128131

[fsn371072-bib-0009] Chen, Q. , H. Qi , X. Zhang , et al. 2017. “SNP–SNP Interaction Analysis of Soybean Protein Content Under Multiple Environments.” Canadian Journal of Plant Science 97, no. 6: 1090–1099.

[fsn371072-bib-0010] Chen, X. , Y. Wang , W. Li , et al. 2015. “Impact of Long‐Term Continuous Soybean Cropping on Ammonia Oxidizing Bacteria Communities in the Rhizosphere of Soybean in Northeast China.” Plant Science 65, no. 5: 470–478.

[fsn371072-bib-0011] Cho, C. H. , Y. S. Jung , T. G. Nam , et al. 2020. “pH‐Adjusted Solvent Extraction and Reversed‐Phase HPLC Quantification of Isoflavones From Soybean (*Glycine max* (L.) Merr.).” Journal of Food Science 85, no. 3: 673–681. 32078761 10.1111/1750-3841.15051

[fsn371072-bib-0012] Choi, J. , E. Noh , D. Lee , Y. Lee , and K. G. Lee . 2023. “Effect of Roasting After Sugar‐Soaking on the Level of Volatile Compounds, Total Polyphenol, Total Flavonoid, and Isoflavones in Black Soybean ( *Glycine max* (L.) Merr.).” LWT ‐ Food Science and Technology 185: 115166.

[fsn371072-bib-0013] Choi, Y. M. , H. Yoon , S. Lee , et al. 2020. “Isoflavones, Anthocyanins, Phenolic Content, and Antioxidant Activities of Black Soybeans ( *Glycine max* (L.) Merrill) as Affected by Seed Weight.” Scientific Reports 10, no. 1: 19960. 33203918 10.1038/s41598-020-76985-4 PMC7673111

[fsn371072-bib-0014] Choi, Y. M. , H. Yoon , M. J. Shin , et al. 2021. “Metabolite Contents and Antioxidant Activities of Soybean ( *Glycine max* (L.) Merrill) Seeds of Different Seed Coat Colors.” Antioxidants 10, no. 8: 1210. 34439461 10.3390/antiox10081210 PMC8388989

[fsn371072-bib-0015] Chu, H. N. , S. J. Lee , X. Wang , et al. 2021. “A Correlation Study on In Vitro Physiological Activities of Soybean Cultivars, 19 Individual Isoflavone Derivatives, and Genetic Characteristics.” Antioxidants 10, no. 12: 2027. 34943130 10.3390/antiox10122027 PMC8698514

[fsn371072-bib-0016] Chung, I. M. , S. H. Seo , J. K. Ahn , and S. H. Kim . 2011. “Effect of Processing, Fermentation, and Aging Treatment to Content and Profile of Phenolic Compounds in Soybean Seed, Soy Curd and Soy Paste.” Food Chemistry 127, no. 3: 960–967. 25214084 10.1016/j.foodchem.2011.01.065

[fsn371072-bib-0017] Costa, G. R. , N. D. O. C. e Silva , J. M. G. Mandarino , et al. 2015. “Isoflavone and Mineral Content in Conventional and Transgenic Soybean Cultivars.” American Journal of Plant Sciences 6, no. 13: 2051–2059.

[fsn371072-bib-0018] de Barros, É. A. , F. Broetto , D. F. Bressan , M. M. Sartori , and V. E. Costa . 2014. “Chemical Composition and Lipoxygenase Activity in Soybeans (*Glycine max* L. Merr.) Submitted to Gamma Irradiation.” Radiation Physics and Chemistry 98: 29–32.

[fsn371072-bib-0019] Epie, K. E. , P. J. Bauer , K. C. Stone , and A. M. Locke . 2023. “Density, Not Tillage, Increases Soybean Protein Concentration in Some Southeastern US Environments.” Agronomy Journal 115, no. 4: 1867–1876.

[fsn371072-bib-0020] González‐Gordo, S. , J. López‐Jaramillo , J. M. Palma , and F. J. Corpas . 2023. “Soybean ( *Glycine max* L.) Lipoxygenase 1 (LOX 1) is Modulated by Nitric Oxide and Hydrogen Sulfide: An In Vitro Approach.” International Journal of Molecular Sciences 24, no. 9: 8001. 37175708 10.3390/ijms24098001 PMC10178856

[fsn371072-bib-0021] Guo, Z. , J. Zhang , C. Ma , et al. 2023. “Application of Visible‐Near‐Infrared Hyperspectral Imaging Technology Coupled With Wavelength Selection Algorithm for Rapid Determination of Moisture Content of Soybean Seeds.” Journal of Food Composition and Analysis 116: 105048.

[fsn371072-bib-0022] Jansone, I. , V. Sterna , V. Stramkale , A. Stramkalis , A. Justs , and S. Zute . 2021. “Impact of Cultivation Technologies on Soybean Production and Quality [Conference Presentation].” Environment Technologies Resources Proceedings of the International Scientific and Practical Conference 1: 101–107.

[fsn371072-bib-0023] Jarecki, W. , and D. Bobrecka‐Jamro . 2021. “Effect of Sowing Date on the Yield and Seed Quality of Soybean ( *Glycine max* (L.) Merr.).” Journal of Elementology 26, no. 1: 7–18.

[fsn371072-bib-0024] Jiao, Z. , X. Si , Z. Zhang , G. Li , and Z. Cai . 2012. “Compositional Study of Different Soybean ( *Glycine max* L.) Varieties by 1H NMR Spectroscopy, Chromatographic and Spectrometric Techniques.” Food Chemistry 135, no. 1: 285–291.

[fsn371072-bib-0025] Josipović, A. , R. Sudar , A. Sudarić , V. Jurković , M. Matoša Kočar , and A. Markulj Kulundžić . 2016. “Total Phenolic and Total Flavonoid Content Variability of Soybean Genotypes in Eastern Croatia.” Czech Journal of Food Sciences 8, no. 2: 60–65.

[fsn371072-bib-0026] Jung, Y. S. , C. S. Rha , M. Y. Baik , N. I. Baek , and D. O. Kim . 2020. “A Brief History and Spectroscopic Analysis of Soy Isoflavones.” Food Science 29: 1605–1617. 10.1007/s10068-020-00815-6 PMC7708537 33282429

[fsn371072-bib-0027] Kamboj, R. , and V. Nanda . 2018. “Proximate Composition, Nutritional Profile and Health Benefits of Legumes—A Review.” Legume Research 41, no. 3: 325–332.

[fsn371072-bib-0028] Kassem, M. A. 2021. “Environmental Factors Affecting Isoflavone Contents.” In Soybean Seed Composition, Oil, Fatty Acids, Amino Acids, Sugars, Mineral Nutrients, Tocopherols, and Isoflavones, 497–511. Springer.

[fsn371072-bib-0029] Khosravi, A. , and S. H. Razavi . 2021. “Therapeutic Effects of Polyphenols in Fermented Soybean and Black Soybean Products.” Journal of Functional Foods 81: 104467.

[fsn371072-bib-0030] Kim, E. H. , S. W. Oh , S. Y. Lee , H. Y. Park , Y. Y. Kang , and G. M. Lee . 2021. “Comparison of the Seed Nutritional Composition Between Conventional Varieties and Transgenic Soybean Overexpressing Physaria FAD3‐1.” Journal of Agricultural and Food Chemistry 101, no. 6: 2601–2613. 10.1002/jsfa.11028 PMC8048611 33336790

[fsn371072-bib-0031] Kudełka, W. , M. Kowalska , and M. Popis . 2021. “Quality of Soybean Products in Terms of Essential Amino Acids Composition.” Molecules 26, no. 16: 5071. https://www.mdpi.com/1420‐3049/26/16/5071. 34443659 10.3390/molecules26165071 PMC8398613

[fsn371072-bib-0032] Kulprachakarn, K. , S. Chaipoot , R. Phongphisutthinant , et al. 2021. “Antioxidant Potential and Cytotoxic Effect of Isoflavones Extract From Thai Fermented Soybean (Thua‐Nao).” Molecules 26, no. 24: 7432. 34946514 10.3390/molecules26247432 PMC8705088

[fsn371072-bib-0033] Lee, H. , B. K. Cho , M. S. Kim , et al. 2013. “Prediction of Crude Protein and Oil Content of Soybeans Using Raman Spectroscopy.” Analytica Chimica Acta 185: 694–700.

[fsn371072-bib-0034] Lei, L. , S. Hui , Y. Chen , H. Yan , J. Yang , and S. Tong . 2024. “Effect of Soy Isoflavone Supplementation on Blood Pressure: A Meta‐Analysis of Randomized Controlled Trials.” Nutrition Journal 23, no. 1: 32. 38454401 10.1186/s12937-024-00932-6 PMC10918941

[fsn371072-bib-0035] Leksawasdi, N. , S. Taesuwan , T. Prommajak , et al. 2022. “Ultrasonic Extraction of Bioactive Compounds From Green Soybean Pods and Application in Green Soybean Milk Antioxidants Fortification.” Food 11, no. 4: 588. 10.3390/foods11040588 PMC8871011 35206064

[fsn371072-bib-0036] Ma, Y. , P. Wang , Z. Gu , M. Sun , and R. Yang . 2022. “Effects of Germination on Physio‐Biochemical Metabolism and Phenolic Acids of Soybean Seeds.” Journal of Food Composition and Analysis 112: 104717.

[fsn371072-bib-0037] McClain, S. , S. E. Stevenson , C. Brownie , et al. 2018. “Variation in Seed Allergen Cont Ent From Three Varieties of Soybean Cultivated in Nine Different Locations in Iowa, Illinois, and Indiana.” Frontiers in Plant Science 9: 1025. 30083174 10.3389/fpls.2018.01025 PMC6065051

[fsn371072-bib-0038] Megha, P. , V. Ramnath , K. Karthiayini , V. Beena , K. Vishnudas , and P. Sapna . 2024. “Free Isoflavone (Daidzein and Genistein) Content in Soybeans, Soybean Meal and Dried Soy Hypocotyl Sprout Using High Performance Liquid Chromatography (HPLC).” Journal of Scientific Research and Reports 30, no. 5: 803–812.

[fsn371072-bib-0039] Mitharwal, S. , and K. Chauhan . 2022. “Impact of Germination on the Proximate Composition, Functional Properties, and Structural Characteristics of Black Soybean (*Glycine max* L. Merr).” Journal of Food Processing and Preservation 46, no. 12: e17202.

[fsn371072-bib-0040] Nemzer, B. V. , F. Al‐Taher , A. Yashin , I. Revelsky , and Y. J. M. Yashin . 2022. “Cranberry: Chemical Composition, Antioxidant Activity and Impact on Human Health: Overview.” Molecules 27, no. 5: 1503. 35268605 10.3390/molecules27051503 PMC8911768

[fsn371072-bib-0041] Obua, T. , J. P. Sserumaga , B. Awio , et al. 2021. “Multi‐Environmental Evaluation of Protein Content and Yield Stability Among Tropical Soybean Genotypes Using GGE Biplot Analysis.” Agronomy 11, no. 7: 1265.

[fsn371072-bib-0042] Özcan, M. M. , and F. Al Juhaimi . 2014. “Effect of Sprouting and Roasting Processes on Some Physico‐Chemical Properties and Mineral Contents of Soybean Seed and Oils.” Food Chemistry 154: 337–342. 24518351 10.1016/j.foodchem.2013.12.077

[fsn371072-bib-0043] Prvulović, D. , Đ. Malenčić , and J. Miladinović . 2016. “Antioxidant Activity and Phenolic Content of Soybean Seeds Extracts.” Acta Agriculturae Serbica 17, no. 2: 121–132.

[fsn371072-bib-0044] Qin, P. , T. Wang , and Y. Luo . 2022. “A Review on Plant‐Based Proteins From Soybean: Health Benefits and Soy Product Development.” Journal of Agriculture and Food Research 7: 100265.

[fsn371072-bib-0045] Raza, H. , Z. Ahmed , M. Hassan , S. Sarwar , S. Saleem , and S. Shazad . 2023. “Assessment of Genetic Variability Among Soybean Exotic Genotypes for Adaptability and Yield.” Crop Science Research 2023, no. 1: 298.

[fsn371072-bib-0046] Rizzo, J. , M. Min , S. Adnan , et al. 2023. “Soy Protein Containing Isoflavones Improves Facial Signs of Photoaging and Skin Hydration in Postmenopausal Women: Results of a Prospective Randomized Double‐Blind Controlled Trial.” Nutrients 15, no. 19: 4113. 37836398 10.3390/nu15194113 PMC10574417

[fsn371072-bib-0047] Saerens, W. , S. Smetana , L. Van Campenhout , V. Lammers , and V. Heinz . 2021. “Life Cycle Assessment of Burger Patties Produced With Extruded Meat Substitutes.” Journal of Cleaner Production 306: 127177.

[fsn371072-bib-0048] Sharma, S. , M. Kaur , R. Goyal , and B. Gill . 2014. “Physical Characteristics and Nutritional Composition of Some New Soybean (*Glycine max* (L.) Merrill) Genotypes.” Journal of Food Science and Technology 51: 551–557. 24587531 10.1007/s13197-011-0517-7 PMC3931876

[fsn371072-bib-0049] Shurtleff, W. , and A. Aoyagi . 2016. History of Modern Soy Protein Ingredients‐Isolates, Concentrates, and Textured Soy Protein Products (1911–2016): Extensively Annotated Bibliography and Sourcebook. Soyinfo Center.

[fsn371072-bib-0050] Staniak, M. , K. Czopek , A. Stępień‐Warda , A. Kocira , and M. Przybyś . 2021. “Cold Stress During Flowering Alters Plant Structure, Yield and Seed Quality of Different Soybean Genotypes.” Agriculture 11, no. 10: 2059.

[fsn371072-bib-0051] Wang, S. Y. , Y. J. Zhang , G. Y. Zhu , et al. 2022. “Occurrence of Isoflavones in Soybean Sprouts and Strategies to Enhance Their Content: A Review.” Journal of Food Science 87, no. 5: 1961–1982. 35411587 10.1111/1750-3841.16131

[fsn371072-bib-0052] Yu, X. , M. Meenu , B. Xu , and H. Yu . 2021. “Impact of Processing Technologies on Isoflavones, Phenolic Acids, and Antioxidant Capacities of Soymilk Prepared From 15 Soybean Varieties.” Food Chemistry 345: 128612. 33352407 10.1016/j.foodchem.2020.128612

[fsn371072-bib-0053] Zhao, Q. , Y. Xu , and Y. Liu . 2022. “Soybean Oil Bodies: A Review on Composition, Properties, Food Applications, and Future Research Aspects.” Food Hydrocolloids 124: 107296.

[fsn371072-bib-0054] AOAC International . 2016. Official Methods of Analysis of AOAC International, 20th Edition. Rockville, MD, USA: AOAC International.

